# Sugar guidelines should be evidence-based and contain simple and easily actionable messages

**DOI:** 10.3389/fnut.2023.1227377

**Published:** 2023-08-15

**Authors:** Rina Ruolin Yan, Jimmy Chun Yu Louie

**Affiliations:** ^1^School of Biological Sciences, Faculty of Science, The University of Hong Kong, Hong Kong, Hong Kong SAR, China; ^2^Department of Nursing and Allied Health, School of Health Sciences, Swinburne University of Technology, Hawthorn, VIC, Australia

**Keywords:** free sugars, guidelines, evidence-based, general public, added sugars

## 1. Introduction

High intakes of added/free sugars have been identified as a major contributor to the current obesity epidemic (1). In response, various public health agencies worldwide, such as the WHO (2), U.S. Department of Agriculture (USDA) (3), and Scientific Advisory Committee on Nutrition (SACN) (4), have issued quantitative guidelines to limit added/free sugars intake to below 5–10% of daily energy intake for improved health (see Supplementary Table 1). While these guidelines have gained acceptance among public health practitioners and researchers, some have raised concerns about their validity, including our research group (5–7). Unfortunately, such skepticism has led to accusations of undermining public health (8). To avoid misinterpretation, we want to clarify that we recognize the need to reduce added/free sugar intake. However, based on the current evidence, we believe the focus should primarily be on reducing sugars from specific sources, such as sugar-sweetened beverages (SSBs), rather than applying the current quantitative guidelines to all food types. In the following sections, we will outline and discuss the rationale behind our skepticism regarding the issuance of quantitative guidelines for added/free sugars intake in the general population.

## 2. Strong evidence supports reducing sugars from SSBs, but not all food sources

Undoubtedly, a substantial body of evidence consistently links sugars from SSBs to adverse health outcomes (9, 10). However, most governments and public health agencies have extended these findings beyond their scope and issued guidelines advocating for a reduction in added/free sugar intake from all food sources (2–4). Nevertheless, studies investigating the effects of sugars from solid foods on metabolic and endocrine health have generally yielded inconclusive results (11–13). For example, while a high intake of liquid sugars has been associated with higher body mass index (BMI) and waist circumference, no such associations have been found for solid sugars in prospective cohort studies involving children (14, 15). Moreover, only a high intake of sugars from liquid sources, not solid foods, has been linked to an increased risk of all-cause mortality (11). In the Swedish prospective cohort study by Ramne et al. (16), high added sugar intake from SSBs was associated with increased all-cause mortality, whereas sugar intake from solid foods was associated with decreased mortality risk.

Studies examining the relationship between sugars from solid foods and the risks of cardiovascular disease (CVD) and type 2 diabetes mellitus (T2DM) have also failed to find a positive association (12, 13). Differential health effects of liquid *vs*. solid sugar sources have been demonstrated in several clinical trials (17, 18). Furthermore, a recent systematic review and meta-analysis that investigated the association of both solid and liquid sources of sugar with the incidence of metabolic syndrome (MetSyn) concluded that only high consumption of SSBs was associated with an increased risk of MetSyn, while no association was found between solid sugar-containing foods like ice cream and confectionery and the incidence of MetSyn (19). The systematic review and meta-analysis of controlled feeding trials by Chiavaroli et al. (20) also concluded that while solid sources of fructose-containing sugars generally have no effects on adiposity irrespective of energy control levels, i.e., substitution (energy-matched replacement of sugars), addition (energy from sugars added), subtraction (energy from sugars removed), and *ad libitum* (energy from sugars freely replaced), with some food sources even leading to decreases in adiposity, e.g., fruits at doses of ≤ 10% daily energy or 50 g/day, excess energy intake from SSBs at ≥ 20% daily energy or 100 g/day leads to increased adiposity. Similarly, Sundborn et al. (21) suggested that liquid sources of added sugar may confer a greater risk of developing MetSyn than solid sources.

## 3. Not all added/free sugars are equal

Our research group has previously discussed the physiological differences between sugars derived from solid and liquid sources (7). Intake of sugars from solid sources is less likely to result in overconsumption of dietary energy (a key contributor to weight gain) due to incomplete compensation for the energy provided by sugars. This disparity may be attributed to the faster gastric emptying time of liquid sugar sources, leading to a higher fructose absorption rate and increased liver exposure to dietary fructose (21, 22). This explanation aligns with findings from animal studies, where the administration of sugar in drinking water led to obesity and metabolic disturbances, while a solid high-sugar diet did not have the same effect (23).

Additionally, some studies have suggested that high intake of SSBs may contribute to overeating and weight gain by disrupting the production of appetite control hormones (24). However, no such effect has been observed with high consumption of solid sugar-containing foods, although the precise mechanisms remain unclear (25). For instance, a study that randomized normal-weight subjects to consume beverages sweetened with fructose or glucose at 30% of their daily energy intake found that the fructose-sweetened group exhibited significantly lower leptin secretion (a hormone that suppresses hunger and appetite) and reduced suppression of circulating ghrelin (a hormone that stimulates appetite and triggers hunger) compared to the glucose-sweetened group (26). Another study showed that consuming fructose-sweetened beverages at 25% of daily energy intake decreased the 24-h leptin area under the curve (AUC) compared to sucrose-sweetened beverages. Interestingly, in a 24-h cross-over study by Stanhope et al. (27) that randomized subjects to consume beverages sweetened with sucrose, high-fructose corn syrup (HFCS), glucose, or fructose, no significant differences were found in 24-h leptin and ghrelin AUC between the groups. Furthermore, excessive fructose intake, such as from SSBs, exposes the liver to high concentrations of fructose, increasing the risk of fat accumulation and associated co-morbidities, whereas the small intestine can convert fructose to glucose and other metabolites at low doses (21).

## 4. Unintended adverse consequences of the current guidelines and related policy directions

In response to the WHO guidelines on free sugar, several agencies and governments have developed and/or implemented sugar reduction targets for a wide range of processed foods, including those not traditionally considered discretionary or junk foods (see Supplementary Table 2) (28–32). However, we believe that these measures may have unintended adverse effects. It is indisputable that added/free sugar are prevalent in our food supply (33, 34). Nevertheless, it is essential to acknowledge that many of these added/free sugar serve functions beyond sweetening agents in processed foods (see Supplementary Table 3) (35). These sugars are used for color and flavor formation, providing bulk and texture, and preservation (35). Reducing or eliminating these sugars, which serve purposes beyond sweetening, often requires substituting the lost functions with other ingredients or food additives to maintain the product's organoleptic properties (35).

Supplementary Table 4 provides examples of ingredient lists comparing similar full-sugar and low-sugar products. Lower-sugar products may also be nutritionally inferior in some instances. Data from the FoodSwitch Hong Kong database, compiled by our group (33), revealed higher saturated fat content in lower-sugar yogurts and yogurt drinks compared to “full” sugar varieties (see Supplementary Table 5). Additionally, the combined use of different food additives may pose potential health risks, although the true extent of these risks remains inadequately researched and largely unknown (36). Even in terms of sweetness, sugars can enhance the palatability of otherwise bland but healthy foods, such as rolled oats (7), as acknowledged in the U.S. Dietary Guidelines (3). Often, the reduced sweetness in reformulated lower-sugar products must be compensated for by using non-caloric or low-calorie sweeteners to maintain the desired taste profile (35). Our group has previously demonstrated that non-caloric and low-calorie sweeteners are now present in a significant proportion of non-low-calorie products (37), which may increase exposure in unsuspecting consumers and potentially lead to adverse health outcomes, such as cancer (38). It should be noted that the current risk assessment approach based on total diet studies (39) does not consider non-low-calorie products as a potential source of these non-caloric or low-calorie sweeteners, leading to a significant underestimation of exposure.

Finally, while the consumption of fresh or minimally processed foods, which are naturally low in added/free sugar, is undoubtedly healthier and should be promoted, it is unrealistic to expect individuals, particularly those with busy lifestyles who rely to some extent on processed foods to meet their nutritional needs, to eliminate processed foods in order to adhere to the recommendation of consuming < 10% of daily energy intake from added/free sugar. Our group (40, 41) and others (42) have shown that for the average consumer, excessively reducing added/free sugar, such as below 5% of daily energy intake, as recommended by the WHO (2) and SACN (4), may result in lower intake of essential micronutrients due to the elimination of many nutrient-dense foods that contain added/free sugar from the diet.

## 5. No ready access to essential information for translating the guidelines into practice

Currently, except for the U.S. (43), labeling added/free sugars on packaged foods is not mandatory. This lack of mandatory labeling means that the general public does not have sufficient information to implement the quantitative guidelines effectively (44). Some public health agencies provide an example upper limit of 50 grams of added/free sugar per day based on a 2,000 kcal/day diet (2), and this limit is often cited in popular media (45). However, without nutrition labels indicating the added/free sugar content of the foods they consume, consumers, who already have trouble differentiating between the terms total, added, and free sugar (44), face difficulties in assessing their added/free sugar intake in relation to this numerical limit. Furthermore, this limit is not directly applicable to individuals with caloric requirements above or below 2,000 kcal/day. Specialized food composition databases that provide added/free sugar values are only available in a limited number of countries (46–48), which means that health professionals in other countries have limited means to evaluate their clients' diets against the quantitative guidelines.

## 6. Lack of relevance and applicability to clinical practice

Given the lack of easily accessible information on the added/free sugar content of foods and beverages, the practical advice given to the general public regarding sugar intake often revolves around limiting the consumption of high-sugar foods and beverages such as SSBs and confectionery (49, 50). In this context, it is uncertain how the quantitative guidelines, which recommend limiting the intake of added/free sugar to below 10% (or 5%) of daily energy intake, offer additional clarity beyond the standard advice. As an extreme example, consumers may be unsure whether a diet containing 10% of energy from added/free sugar, primarily from SSBs, is healthier than a diet that contains 15% of energy from added/free sugar derived from a mix of nutrient-dense foods (e.g., breakfast cereals, sweetened yogurt).

## 7. Discussion and final remarks

We believe that guidelines regarding sugars and health for the general public should consist of clear and practical messages that are easily understood and can be implemented. These messages should focus on limiting the consumption of SSBs and other high-sugar discretionary/junk foods, as these recommendations are supported by robust scientific evidence. While there may be ongoing controversies surrounding the validity of quantitative targets for added/free sugar intake, we suggest that such targets be reserved for research purposes. Currently, consumers and health professionals in most parts of the world lack the necessary knowledge and information to apply these quantitative guidelines in their daily lives effectively. Therefore, it is essential to prioritize accessible and actionable recommendations that align with the understanding and needs of the general population.

## Author contributions

JL conceived the idea, wrote the first draft of the article, and has primary responsibility for the content presented. RRY collected information for the tables and figures and contributed substantially to the subsequent writing of the article. All authors contributed to the article and approved the submitted version.
