# Supplementary material for: Sugar guidelines should be evidence-based and contain simple and easily actionable messages
Source: Front Nutr. 2023 Aug 15;10:1227377. doi: 10.3389/fnut.2023.1227377 (PMC10464488; doi:10.3389/fnut.2023.1227377)
Supplement: Supplementary file 1 [file Data_Sheet_1.docx]

Supplementary Material

Sugar guidelines should be evidence-based and contain simple and easily actionable messages

Rina Ruolin Yan, Jimmy Chun Yu Louie^*^

*** Correspondence:** A/Prof Jimmy Chun Yu Louie: [jimmylouie@swin.edu.au](mailto:jimmylouie@swin.edu.au)

**Online Supplemental Table 1** Examples of quantitative added/free sugars intake guidelines around the world

| **Organization/Country** | **Guidelines** |
| --- | --- |
| *WHO* [1] | Reduction of free sugar intake to < 10% (strong recommendation) or 5% (conditional recommendation) of daily energy |
| *USDA* [2] | Limiting added sugar intake to < 10% of daily energy |
| *SACN* [3] | Limiting free sugar intake to < 5% of daily energy |
| *AHA* [4] | Limiting added sugar intake to ≤ 6% of daily energy |
| *CFSA* [5] | Added sugar intake at ≤ 50 g per day, preferably ≤ 25 g |
| *Ministry of Health of Brazil* [6] | Limiting added sugar intake to < 10% of daily energy |

AHA, American Heart Association; CFSA, China National Centre for Food Safety Risk Assessment; SACN, Scientific Advisory Committee on Nutrition; USDA, U.S. Department of Agriculture; WHO, World Health Organization

**Online Supplemental Table 2** – Examples of sugar reduction targets around the world

| **Country** | **Targets** |
| --- | --- |
| **U.S.* [7] | 10% (2023) and 20% (2026) reduction from baseline sales-weighted mean sugar density (grams of sugar per 100 g of food) for sugar-containing solid foods; 10% (2023) and 40% (2026) reduction for sugary drinks from baseline sales-weighted mean sugar density (grams of sugar per 100 mL of beverages) |
| *The European Union and the U.K.* [8] | 10% reduction in calories from added sugars in soft drinks from 2019 to 2025 |
| *Australia* [9] | Muesli and snack bars: maximum 25 g/100 g and at least a 15% reduction for products containing > 28.5 g sugar/100 g by June 2025  Non-alcoholic beverages: maximum 5 g/100 mL by June 2025 |
| *New Zealand* [10] | Savoury snacks: maximum 25 g/100 g or 15% reduction for products with total sugar content significantly > 25 g/100 g (2019-2024)  Flavoured dairy milk: 7.0 g/100 mL or 10% reduction for products with total sugar content significantly > 7.0 g/100 mL (2018-2023) |

U.S., United States; U.K., United Kingdom; UNESDA, Union of European Soft Drinks Associations

*refers to the National Salt and Sugar Reduction Initiative, a partnership of over 100 state and local health authorities and national health organizations from across U.S., convened by the New York City Department of Health and Mental Hygiene (NYC Health Department) to encourage voluntary corporate commitments to sugar and salt reduction targets. These targets are not endorsed nor supported by the U.S. government.

**Online Supplemental Table 3 –** Functions of sugars in foods and functional replacement alternatives

| **Functions** | **Alternatives** |
| --- | --- |
| *Sweetness* | Non-nutritive or low-calorie sweeteners such as maltitol and sucralose |
| *Water retention* | Humectants and stabilizers such as sorbitol and maltitol |
| *Colour formation* | Artificial and natural food colourants |
| *Flavour (enhancement)* | Natural and artificial flavouring, flavour enhancers, and non-nutritive or low-calorie sweeteners such as maltitol, isomalt, and sucralose |
| *Fermentation* | Leavening agents and acids such as sodium bicarbonate |
| *Texture and bulk* | Gels, gums, starches, and stabilizers such as xanthan gum, modified starch, and agar |
| *Preservation* | Preservatives such as organic acids and antioxidants |

Table adapted from Goldfein and Slavin [11], Jorge [12], Petković [13], Stich [14], Saraiva *et al.* [15], Zeece [16], Khubber *et al.* [17], da Silva Costa *et al.* [18], Saha and Bhattacharya [19], and García-García and Searle [20]

**Online Supplemental Table 4 –** Examples of ingredients lists for full sugar *vs.* low sugar products

|  | **Full sugar version** | **Low sugar version** |
| --- | --- | --- |
| *Strawberry yoghurt* | ^a^Cultured Grade A Low Fat Milk, Sugar, Strawberries, Modified Food Starch, Water. Contains 1% or less of: Corn Starch, Tricalcium Phosphate, Carmine (for color), Pectin, Natural Flavor, Kosher Gelatin, Vitamin A Acetate, Vitamin D_3_. | ^b^Cultured Grade A Nonfat Milk, Strawberries, Water, Modified Corn Starch, **Allulose**, **Kosher Gelatin**, Citric Acid, Tricalcium Phosphate, Natural Flavor, **Sucralose**, **Potassium Sorbate**, **Acesulfame Potassium**, Red #40, Vitamin A Acetate, Vitamin D_3_. |
| *Chocolate chip cookies* | ^c^Wheat Flour (Wheat Flour, Calcium Carbonate, Niacin, Iron, Thiamin), Chocolate Chips (25%) (Sugar, Cocoa Mass, Vegetable Fats (Sustainable Palm, Shea, Sal), Emulsifiers (Soya Lecithin, E442, E476), Cocoa Butter, Flavourings), Sugar, Sustainable Palm Oil, Whey or Whey Derivatives (Milk), Partially Inverted Sugar Syrup, Raising Agents (Sodium Bicarbonate, Ammonium Bicarbonate), Salt, Flavourings | ^d^Wheat Flour (Wheat Flour, Calcium Carbonate, Niacin, Iron, Thiamin), Chocolate Chips with Sweetener (20%) (**Sweetener (Maltitol)**, Cocoa Mass, Emulsifier (Soya Lecithin), Fat Reduced Cocoa Powder, Flavouring), **Sweetener (Maltitol)**, Sustainable Palm Oil, Flavourings, Raising Agents (Sodium Bicarbonate, Disodium Diphosphate, Ammonium Bicarbonate), Salt, Colour (Paprika Extract) |
| *Caramel snack bar* | ^e^Milk Chocolate (40%)(Sugar, Cocoa Butter**, Dried Whole Milk, Cocoa Mass**, Milk Sugar, Sweet Whey Powder (Milk), Emulsifier (Soya Lecithins), Salt, Vanilla Extract), Sugar, Glucose Syrup, Palm Fat, Condensed Skimmed Milk, Barley Malt Extract, Dried Skimmed Milk, Fat Reduced Cocoa Powder**, Caramelised Sugar Syrup, Salt, Dried Egg White | ^f^Milk Chocolate with Sweetener 20.8% (**Sweetener (Maltitol)**, Cocoa Butter, Whole Milk Powder, Cocoa Mass, Emulsifier (Soy Lecithin), Flavourings), Caramel Flavoured Layer 15% (**Bulking Agent (Polydextrose)**, Soy Oil, **Sweeteners (Xylitol, Sucralose)**, Skimmed Milk Powder, Soy Protein, Flavourings, Emulsifier (Soy Lecithin), Salt), **Humectant (Glycerol)**, Soy Protein, **Bulking Agent (Polydextrose)**, Hydrolysed Wheat Gluten, Milk Protein, **Hydrolysed Collagen**, Cocoa Butter, Milk Fat, Sunflower Oil, Cocoa Mass, Emulsifier (Soy Lecithin), Flavourings, **Sweetener (Sucralose)** |

Ingredients used to replace sugar’s function are highlighted in bold

^a^<https://www.yoplait.com/products/original-single-serve-strawberry>

^b^<https://www.yoplait.com/products/light-single-serve-strawberry>

^c^<https://www.tesco.com/groceries/en-GB/products/293418761>

^d^<https://www.tesco.com/groceries/en-GB/products/311952250>

^e^<https://www.tesco.com/groceries/en-GB/products/300876915>

^f^<https://www.tesco.com/groceries/en-GB/products/309781879>

**Online Supplemental Table 5** – The nutritional profile of low (< 5 g per 100 g/100 mL) *vs*. high (≥ 5 g per 100 g/ 100 mL) sugar yogurt and yogurt drinks

| **Nutrient (per 100 g or 100 mL)** | **Low sugar products** | **High sugar products** | ***p* value** |
| --- | --- | --- | --- |
| *n* | 48 | 281 | - |
| Energy (kJ) | 345 ± 182 | 413 ± 218 | 0.042 |
| Protein (g) | 4.8 ± 2.6 | 3.8 ± 1.6 | < 0.001 |
| Total fat (g) | 4.9 ± 5.2 | 2.8 ± 2.4 | < 0.001 |
| Saturated fat (g) | 3.4 ± 4.5 | 1.8 ± 1.8 | < 0.001 |
| *Trans-*fat (g) | 0.1 ± 0.2 | 0.0 ± 0.1 | 0.033 |
| Carbohydrates (g) | 4.9 ± 1.4 | 13.9 ± 10.1 | < 0.001 |
| Total sugar (g) | 3.2 ± 1.6 | 12.0 ± 5.9 | < 0.001 |
| Free sugar (g) | 0.1 ± 0.5 | 7.2 ± 6.1 | < 0.001 |
| Fibre (g) | 0.6 ± 0.8 | 0.7 ± 0.8 | 0.630 |
| Sodium (mg) | 49.3 ± 29.2 | 86.1 ± 534.8 | 0.634 |
| ^a^Health Star Rating | 3.7 ± 1.5 | 2.9 ± 1.1 | < 0.001 |

^a^The Health Star Rating is a front-of-package nutritional rating system used by the Australian government, with possible points from 0.5 to 5.0 in 0.5 points increments

**References**

[1] World Health Organization, Guideline: Sugar intake for adults and children, WHO Department of Nutrition for Health and Development (NHD), Geneva, Switzerland, 2015, pp. 50.

[2] U.S. Department of Agriculture, Dietary Guidelines for Americans 2020 - 2025, USDA, Washington D.C., USA, 2020.

[3] Scientific Advisory Committee on Nutrition, Why 5%? An explanation of the Scientific Advisory Committee on Nutrition’s recommendations about sugars and health, in the context of current intakes of free sugars, other dietary recommendations and the changes in dietary habits needed to reduce consumption of free sugars to 5% of dietary energy, Public Health England, London, UK, 2015.

[4] American Heart Association, Added sugars, 2021.

[5] China National Centre for Food Safety Risk Assessment, Sugar intake and risk assessment in Chinese urban residents, 2016.

[6] Ministry of Health of Brazil, Dietary guidelines for the Brazilian population, 2015.

[7] New York City Department of Health and Mental Hygiene, Sugar reduction targets from the national salt and sugar reduction initiative, 2021.

[8] Union of European Soft Drinks Associations, Soft drinks industry's sugar reduction commitments around Europe, 2022.

[9] Australian Government Department of Health and Aged Care, Partnership reformulation program, 2022.

[10] Heart Foundation, Heart Foundation food reformulation targets, 2020.

[11] K.R. Goldfein, and J.L. Slavin, Why Sugar Is Added to Food: Food Science 101. Comprehensive Reviews in Food Science and Food Safety 14 (2015) 644-656.

[12] K. Jorge, SOFT DRINKS | Chemical Composition. in: B. Caballero, (Ed.), Encyclopedia of Food Sciences and Nutrition (Second Edition), Academic Press, Oxford, 2003, pp. 5346-5352.

[13] M. Petković, Alternatives for sugar replacement in food technology: formulating and processing key aspects. in: T.E. Coldea, (Ed.), Food Engineering, Intechopen, London, 2019.

[14] E. Stich, 1 - Food Color and Coloring Food: Quality, Differentiation and Regulatory Requirements in the European Union and the United States. in: R. Carle, and R.M. Schweiggert, (Eds.), Handbook on Natural Pigments in Food and Beverages, Woodhead Publishing, 2016, pp. 3-27.

[15] A. Saraiva, C. Carrascosa, D. Raheem, F. Ramos, and A. Raposo, Natural sweeteners: the relevance of food naturalness for consumers, food security aspects, sustainability and health impacts. Int. J. Environ. Res. Public Health 17 (2020).

[16] M. Zeece, Chapter One - Chemical properties of water and pH. in: M. Zeece, (Ed.), Introduction to the chemistry of food, Academic Press, Cambridge, 2020, pp. 1-36.

[17] S. Khubber, F.J. Marti-Quijal, I. Tomasevic, F. Remize, and F.J. Barba, Lactic acid fermentation as a useful strategy to recover antimicrobial and antioxidant compounds from food and by-products. Curr. Opin. Food Sci. 43 (2022) 189-198.

[18] R.A. da Silva Costa, R.C.F. Bonomo, L.B. Rodrigues, L.S. Santos, and C.M. Veloso, Improvement of texture properties and syneresis of arrowroot (Maranta arundinacea) starch gels by using hydrocolloids (guar gum and xanthan gum).

J. Sci. Food Agric. 100 (2020) 3204-3211.

[19] D. Saha, and S. Bhattacharya, Hydrocolloids as thickening and gelling agents in food: a critical review. J. Food Sci. Technol. 47 (2010) 587-97.

[20] R. García-García, and S.S. Searle, Preservatives: Food Use. in: B. Caballero, P.M. Finglas, and F. Toldrá, (Eds.), Encyclopedia of Food and Health, Academic Press, Oxford, 2016, pp. 505-509.
